# Variable phenotypes and outcomes associated with the *MMACHC* c.482G > A mutation: follow-up in a large CblC disease cohort

**DOI:** 10.1007/s12519-023-00770-2

**Published:** 2023-12-09

**Authors:** Sheng-Nan Wu, Hui-Shu E, Yue Yu, Shi-Ying Ling, Li-Li Liang, Wen-Juan Qiu, Hui-Wen Zhang, Rui-Xue Shuai, Hai-Yan Wei, Chi-Ju Yang, Peng Xu, Xi-Gui Chen, Hui Zou, Ji-Zhen Feng, Ting-Ting Niu, Hai-Li Hu, Kai-Chuang Zhang, De-Yun Lu, Zhu-Wen Gong, Xia Zhan, Wen-Jun Ji, Xue-Fan Gu, Yong-Xing Chen, Lian-Shu Han

**Affiliations:** 1 Department of Endocrinology and Metabolism, Henan Key Laboratory of Children’s Genetics and Metabolic Diseases, Children’s Hospital Affiliated to Zhengzhou University, Henan Children’s Hospital, Zhengzhou Children’s Hospital, No. 255 Gangdu Street, Zhengzhou, China; 2 Department of Pediatric Endocrinology and Genetics, Fujian Children’s Hospital, Fuzhou, China; 3 The Center for Pediatric Liver Diseases, Children’s Hospital, Fudan University, Shanghai, China; 4 Department of Pediatric Endocrinology/Genetics, Shanghai Institute for Pediatric Research, Xinhua Hospital, School of Medicine, Shanghai Jiao Tong University, No. 1665 Kongjiang Road, Shanghai, China; 5 Department of Pediatrics, Second Affiliated Hospital of Naval Medical University, Shanghai, China; 6 Center of Neonatal Disease Screening, Jining Maternal and Child Health Care Hospital, Jining, China; 7 Center of Neonatal Disease Screening, Jinan Maternal and Child Health Care Hospital, Jinan, China; 8 Center of Neonatal Disease Screening, Shijiazhuang Maternal and Child Health Care Hospital, Shijiazhuang, China; 9 Center of Neonatal Disease Screening, Shandong Maternal and Child Health Care Hospital, Jinan, China; 10 Center of Neonatal Disease Screening, Hefei Maternal and Child Health Care Hospital, Hefei, China

**Keywords:** c.482G > A, CblC disease, *MMACHC* gene, Newborn screening, Outcome

## Abstract

**Background:**

The aim of this study was to characterize the variable phenotypes and outcomes associated with the methylmalonic aciduria and homocystinuria type C protein gene (*MMACHC*) c.482G > A mutation in 195 Chinese cases with CblC disease.

**Methods:**

We carried out a national, retrospective multicenter study of 195 Chinese patients with CblC disease attributable to the *MMACHC* c.482G > A variant either in a homozygous or compound heterozygous state. The control group consisted of 200 patients diagnosed with CblC disease who did not possess the c.482G > A mutation. Clinical features, including disease onset, symptoms, biochemical metabolites, gene mutation, and follow-up outcomes were reviewed and analyzed in detail. The median follow-up period spanned 3 years and 8 months, with a range of 1 year and 2 months to 12 years and 10 months.

**Results:**

Among 195 patients carrying the c.482G > A variant, 125 (64.1%) cases were diagnosed by newborn screening (NBS), 60 (30.8%) cases were detected due to disease onset, and 10 (5.1%) cases were identified from sibling diagnoses. One hundred and seventeen (93.6%) individuals who were diagnosed by NBS, and nine patients who came from sibling diagnoses remained asymptomatic in this study. From 69 symptomatic patients of the c.482G > A group, more patients presented with later onset, and the top six common clinical symptoms at disease onset were developmental delay (59.4%), lower limb weakness and poor exercise tolerance (50.7%), cognitive decline (37.7%), gait instability and abnormal posture (36.2%), seizures (26.1%), and psychiatric and behavioral disturbances (24.6%). In the 159 symptomatic patients lacking c.482G > A variants, the most frequently observed clinical manifestations at disease onset included developmental delay (81.8%), lethargy and feeding difficulty (62.9%), lower limb weakness and poor exercise tolerance (54.7%), prolonged neonatal jaundice (51.6%), vomiting (47.2%), and seizures (32.7%). Before treatment, the levels of blood propionylcarnitine, propionylcarnitine/acetylcarnitine ratio, and homocysteine in the c.482G > A group were significantly lower (*P* < 0.05) than those in the non-c.482G > A group, while the concentration of urinary methylmalonic acid was slightly lower (*P* > 0.05). The degree of decline in the above metabolites after treatment in different groups significantly differed in both plasma total homocysteine values and urinary methylmalonic acid levels (*P* < 0.05). In patients carrying the c.482G > A variant compared with the non-c.428G > A group, there were markedly lower rates of mortality (0.5% vs. 2.0%) and developmental delay (20.5% vs. 65.5%). When compared with individuals diagnosed due to disease onset, those identified through NBS in either group exhibited a reduced proportion of disease onset (6.7% vs. 100% in the c.482G > A group, 54.4% vs. 100% in the non-c.482G > A group), lower mortality (0.0% vs. 1.7% in the c.482G > A group, 0.0% vs. 3.6% in the non-c.482G > A group), and had a higher percentage of patients exhibiting normal psychomotor and language development (99.3% vs. 33.3% in the c.482G > A group, 58.9% vs. 10.9% in the non-c.482G > A group).

**Conclusions:**

The c.482G > A variant in *MMACHC* is associated with late-onset and milder phenotypes of CblC disease. Patients with this mutation tend to have a relatively better response to hydroxocobalamin, better metabolic control, and more favorable neurological outcomes. NBS and other appropriate pre-symptomatic treatments seem to be helpful in early diagnosis, resulting in favorable clinical outcomes.

Video Abstract (MP4 136794 kb)

**Supplementary Information:**

The online version contains supplementary material available at 10.1007/s12519-023-00770-2.

## Introduction

The CblC type of combined methylmalonic acidemia (MMA) and homocysteinemia (CblC disease) caused by mutations in the *MMACHC* gene is the most common defect in the intracellular cobalamin metabolism pathway, which is also the most common type of MMA in China [1, 2]. This defect can result in impaired synthesis of adenosylcobalamin and methylcobalamin, which, respectively, are essential coenzymes for the conversion of methylmalonyl-CoA into succinyl-CoA (catalyzed by methylmalonyl-CoA mutase) and remethylation of homocysteine to methionine (catalyzed by methionine synthase). According to the age of onset, patients can be divided into two types, early-onset form (onset before the age of 1 year) and late-onset form (onset after the age of 4 years) [3, 4], exhibiting differing genotypes and severity of disease. The late-onset CblC disease commonly presents with atypical clinical symptoms, which may lead to misdiagnosed and delayed treatment. Several studies have reported the c.482G > A (p.R161Q) variation in the *MMACHC* gene is related to late-onset or milder disease [5–7]. However, the clinical features and outcome of individuals with CblC disease with the c.482G > A (p.R161Q) mutation are yet to be comprehensively explored.

In the present study, we examined 195 Chinese patients with CblC disease carrying the c.482G > A variant of *MMACHC* and performed a retrospective review of their clinical data. To investigate their clinical features, outcome, and the potential relationship between genotype and phenotype for the specific mutation c.482G > A, we compared these data with those of patients carrying other mutations in the *MMACHC* gene. To our knowledge, this study comprises the largest cohort of patients with the c.482G > A variant.

## Methods

### Patients and clinical data collection

From 2010 to 2021, a total of 2208 patients with MMA were diagnosed at multiple hospitals in China. Among them, 1695 (76.8%) cases were caused by *MMACHC* gene mutations. We searched for cases carrying the c.482G > A mutation in these patients. As a result, 195 (195/1695, 11.5%) patients were assigned to the “c.482G > A group”, most of which were compound heterozygous with missense, nonsense, frameshift variants or exon deletion in combination with c.482G > A. Their clinical characteristics, metabolites, molecular features, and outcomes were reviewed. The median duration of follow-up was 3 years and 8 months, ranging from 1 year and 2 months to 12 years and 10 months. To match the patients carrying c.482G > A and “another mutation” in another allele, we selected a further 200 patients with combined methylmalonic aciduria and homocystinuria without the c.482G > A mutation in the *MMACHC* gene but sharing some “another mutation” with patients in the c.482G > A group, as a paired control group (non-c.482G > A group). We compared the clinical and biochemical phenotypes, and outcomes of patients from the two groups. Informed consent was obtained from the legal guardians of each patient.

### Detection of metabolites

Tandem mass spectrometry was used to evaluate amino acid and acylcarnitine concentrations in dried blood spots using a tandem mass spectrometer (API 4000, American Bio-Systems Inc.), as previously described [8]. Urine organic acid concentrations were detected by gas chromatography and mass spectrometry (GC–MS) (GCMS-QP 2010, Shimadzu Corporation, Kyoto, Japan), using a previously established protocol [9]. Moreover, plasma total homocysteine (HCY) was assessed using a fluorescence polarization immunoassay.

### Molecular genetic analysis

Some patients underwent genetic testing in other research institutions. For patients without genetic testing, patients carrying novel mutations or monoallelic variations in the *MMACHC* gene, and all patients diagnosed in our hospital, we collected their samples for Sanger sequencing or high-throughput next-generation sequencing. Genomic DNA was extracted from the peripheral blood of the patients and their parents using a Lab-Aid blood DNA isolation kit (DNA DP319–01; Tiangen Biotech Co. Ltd.) according to the manufacturer’s instructions. *MMACHC* gene detection was performed by Sanger sequencing or high-throughput next-generation sequencing. The raw data were aligned to the normal human *MMACHC* sequence as a reference (NM_015506.3). Variant descriptions were fully reviewed based on the standard human sequence variant nomenclature of the Human Genome Variation Society [10]. The classification of novel variants was evaluated according to the method recommended by the American College of Medical Genetics and Genomics [11]. We used the ClinVar database, the Human Gene Mutation Database, and previous literature to identify whether the mutations had been reported or not.

### Treatment

The treatment of CblC disease varies with different vitamin B12 responsiveness. Generally, for most patients, long-term therapy involved intramuscular injections of hydroxocobalamin at a dose of 1–20 mg each time, once every 1–20 days, L-carnitine at 50–100 mg/kg per day, and oral betaine at 100–300 mg/kg per day. In patients with methionine deficiency, methionine was given as an oral supplement. The treatment was then adjusted depending on the metabolic condition of individual patients [3, 12].

### Follow-up and outcome evaluations

The clinical follow-up outcome evaluation mainly included biochemical results after treatment, assessment of disease onset, as well as basic motor function and language development. Follow-up procedures involved telephone interviews and in-person patient visits. During telephone interviews, parents provided information on their child’s height, weight, motor and/or speech, and cognitive development status. During the follow-up visit, an experienced pediatric clinician assessed the patient. According to the basic motor function and language development evaluation method mentioned in the literature [13], patients were divided into two main groups: normal and delayed. The normal group exhibited no significant impairments in daily functioning, whereas the delayed group demonstrated deficits and/or delayed attainment of motor and/or speech milestones, such as requiring assistance for ambulation and being unable to effectively communicate or articulate words.

### Statistical analysis

SPSS 26.0 (SPSS Inc., Chicago, IL, USA) was used for statistical analyses. Analyses using the Shapiro–Wilk method showed that the data did not conform to normal distribution. Therefore, a nonparametric unpaired Mann–Whitney *U* test was performed. The values were expressed as median (minimum–maximum). The comparison of rates was calculated using a Chi-square test. Multiple comparisons were adopted using the Bonferroni method. The *P* value < 0.05 was considered as a statistically significant difference between the two groups.

## Results

### Patient demographics

Detailed information on patients in the two groups is shown in Table 1. Among the c.482G > A group (118 males, 77 females), a total of 125 (64.1%) affected individuals were identified with MS/MS-based newborn screening (NBS). One hundred and seventeen cases remained asymptomatic at the time of this study with a median age of 3 years and 3 months (range from 11 months to 8 years old). While waiting for the results of NBS, seven cases developed symptoms, respectively, at the age ranged from 1 to 22 days, and then treatment was administered immediately. Their symptoms included vomiting, feeding difficulty, prolonged neonatal jaundice, and lethargy. At the last follow-up, these seven patients were aged 2–6 years old, with normal physical and psychomotor development. Unfortunately, among the 125 cases detected by NBS, one patient was found with developmental delay at the age of 17 months, due to the refusal of confirmative testing and pre-symptomatic treatment.

**Table 1:** General information of patients in different groups

Patients	Diagnosed from newborn screening			Diagnosed due to disease onset	Diagnosed due to sibling diagnosis			Total number of symptomatic patients
	Total number	Symptomatic	Asymptomatic		Total number	Symptomatic	Asymptomatic	
c.482G > A group (*n* = 195)	125/195 (64.1)	8/125 (6.4)	117/125 (93.6)	60/195 (30.8)	10/195 (5.1)	1/10 (10.0)	9/10 (90.0)	69/195 (35.4)
Non-c.482G > A group (*n* = 200)	90/200 (45.0)	49/90 (54.4)	41/90 (45.6)	110/200 (55.0)	0 (0.0)	0 (0.0)	0 (0.0)	159/200 (79.5)

Data are presented as *n/n* (%)

Sixty patients in the c.482G > A group were diagnosed because of disease onset at the median age of 7 years and 6 months (ranging from 1 day to 33 years). Ten cases were diagnosed because of sibling MMA diagnosis. Among these 10 individuals, nine were asymptomatic, while the other one developed lower limb weakness and poor exercise tolerance, and a decline in learning and memory at 9 years of age for unknown reasons. The patient was diagnosed and treated until 4 years later when the younger sibling was diagnosed with MMA through NBS. Fortunately, this patient is currently 16 years old and has no obvious abnormality in motor and language development after reasonable treatment.

In the non-c.482G > A group, which included 107 males and 93 females, a total of 90 individuals were identified through NBS by MS/MS and received timely pre-symptomatic treatment. Of these, 41 cases remained asymptomatic with a median age of 3 years and 2 months (ranging from 2 to 10 years), while 49 cases continued to exhibit a range of symptoms. This included patients who developed symptoms very early, prior to receiving NBS results, and those who became symptomatic despite receiving adequate and timely treatment. The median age of symptom onset was 24 days (ranging from 1 day to 4 years and 9 months). There were 110 patients diagnosed because of the onset of the disease with a median age of onset of 40 days (ranging from 1 hour to 8 years). In general, the proportion of symptomatic patients in the c.482G > A group was significantly lower than that in the non-c.482G > A group (*P* < 0.01), even in patients who were diagnosed with NBS and started treatment on time.

### Clinical manifestations

The clinical characteristics of all symptomatic patients in different groups are summarized in Table 2. More patients presented with later onset in the c.482G > A group than those in the non-c.482G > A group. More than half of patients with a c.482G > A variation developed their first symptom after the age of 4 years. Seven of them showed symptomatic presentation during adulthood (the median age was 22 years, ranging from 19 to 33 years). In the non-c.482G > A group, 80.5% of patients developed their first symptom before the age of 1 year. The difference was statistically significant (*P* < 0.01).

**Table 2:** Clinical characteristics of symptomatic patients in different groups at onset

Common clinical manifestations	c.482G > A group			Non-c.482G > A group			Total patients in different groups	
	Total (*N* = 69)	Diagnosed from NBS and sibling diagnosis (*n* = 8 + 1)	Diagnosed due to disease onset (*n* = 60)	Total (*N* = 159)	Diagnosed from NBS (*n* = 49)	Diagnosed due to disease onset (*n* = 110)	*χ*^2^	*Ρ*
Age of disease onset (y)								
≤ 1	22 (31.9)	8 (88.9)	14 (23.3)	128 (80.5)	40 (81.6)	88 (80.0)	80.738	< 0.050
1–4	10 (14.5)	0 (0.0)	10 (16.7)	25 (15.7)	8 (16.3)	17 (15.5)		
> 4	37 (53.6)	1 (11.1)	36 (60.0)	6 (3.8)	1 (2.0)	5 (4.5)		
Developmental delay^a,b,c^	41 (59.4)	1 (11.1)	40 (66.7)	130 (81.8)	36 (73.5)	94 (85.5)	12.809	< 0.050
Lower limb weakness and poor exercise tolerance	35 (50.7)	1 (11.1)	34 (56.7)	87 (54.7)	20 (40.8)	67 (60.9)	0.308	0.579
Decline in cognitive, learning, and memory^c^	26 (37.7)	1 (11.1)	25 (41.7)	12 (7.5)	1 (2.0)	11 (10.0)	31.460	< 0.050
Gait instability and abnormal posture^c^	25 (36.2)	0 (0.0)	25 (41.7)	19 (11.9)	3 (6.1)	16 (14.5)	18.217	< 0.050
Seizures	18 (26.1)	1 (11.1)	17 (28.3)	52 (32.7)	11 (22.4)	41 (37.3)	0.990	0.320
Psychiatric and behavioral disturbance^c^	17 (24.6)	0 (0.0)	17 (28.3)	8 (5.0)	1 (2.0)	7 (6.4)	18.947	< 0.050
Lethargy^c^	12 (17.4)	2 (22.2)	10 (16.7)	100 (62.9)	27 (55.1)	73 (66.4)	39.862	< 0.050
Feeding difficulty^a,c^	12 (17.4)	5 (55.6)	7 (11.7)	100 (62.9)	27 (55.1)	73 (66.4)	39.862	< 0.050
Prolonged neonatal jaundice^a,c^	8 (11.6)	4 (44.4)	4 (6.7)	82 (51.6)	26 (53.1)	56 (50.9)	32.189	< 0.050
Vomiting^c,d^	5 (7.2)	2 (22.2)	3 (5.0)	75 (47.2)	15 (30.6)	60 (54.5)	33.673	< 0.050
Coma	5 (7.2)	0 (0.0)	5 (8.3)	21 (13.2)	3 (6.1)	18 (16.4)	1.692	0.193
Incontinence	4 (5.8)	0 (0.0)	4 (6.7)	0 (0.0)	0 (0.0)	0 (0.0)	6.320	0.012
Hydrocephalus	3 (4.3)	0 (0.0)	3 (5.0)	16 (10.1)	2 (4.1)	14 (12.7)	2.057	0.151
Renal dysfunction	3 (4.3)	0 (0.0)	3 (5.0)	1 (0.6)	0 (0.0)	1 (0.9)	2.005	0.157
Ocular problems	2 (2.9)	0 (0.0)	2 (3.3)	19 (11.9)	8 (16.3)	11 (10.0)	4.714	0.030
Pulmonary hypertension	1 (1.4)	0 (0.0)	1 (1.7)	1 (0.6)	0 (0.0)	1 (0.9)	0.000	1.000

Data are presented as *n* (%). *NBS* newborn screening. ^a^In the c.482G > A group, there was a statistically significant difference between the patients diagnosed by NBS and those diagnosed after onset; ^b^among the patients diagnosed by NBS, there was a statistically significant difference between the c.482G > A group and non-c.482G > A group; ^c^among the patients diagnosed after onset, there was a statistically significant difference between the c.482G > A group and non-c.482G > A group; ^d^in the non-c.482G > A group, there was a statistically significant difference between the patients diagnosed by NBS and those diagnosed after onset

In 69 symptomatic patients with c.482G > A variant, the most common clinical symptoms at onset were developmental delay (59.4%), lower limb weakness and poor exercise tolerance (50.7%), decline in cognitive, learning, and memory (37.7%), gait instability and abnormal posture (36.2%), seizures (26.1%), and psychiatric and behavioral disturbances (24.6%). Among the seven patients who had disease onset during adulthood, the initial clinical manifestations were psychiatric and behavioral disturbance. On the other hand, in the 159 symptomatic patients without the c.482G > A variant, the most common clinical symptoms at onset were developmental delay (81.8%), lethargy and feeding difficulty (62.9%), lower limb weakness and poor exercise tolerance (54.7%), prolonged neonatal jaundice (51.6%), vomiting (47.2%), and seizures (32.7%). For clinical manifestations that were less common, we observed that ocular problems and hydrocephalus mainly presented in the non-c.482G > A group while incontinence was found in the c.482G > A group only. Pulmonary hypertension was a rare symptom in both groups. Furthermore, compared with patients diagnosed due to disease onset, the proportion of clinical symptoms in individuals identified by NBS displayed no significant differences, with the exception of developmental delay, feeding difficulty, and prolonged neonatal jaundice in the c.482G > A group. In contrast, for the non-c.482G > A group, only the proportion of patients experiencing vomiting exhibited a significant difference between those diagnosed due to disease onset and those identified through NBS.

### Biochemical features

The concentrations of blood propionylcarnitine (C3), propionylcarnitine/acetylcarnitine ratio (C3/C2) and total plasma HCY, and urinary methylmalonic acid values for each group of patients before and after medical intervention are summarized in Table 3. Finally, complete biochemical data both before and after treatment of 149 patients in the c.482G > A group and 150 patients in the non-c.482G > A group were available. In this study, the levels of blood C3, C3/C2 ratio and total plasma HCY, as well as urinary methylmalonic acid levels were above the cutoff values before medical intervention in all patients. Before treatment, levels of these metabolites were lower in patients in the c.482G < A group compared with those in the non-c.482G < A group. Between the two groups, differences in the levels of blood C3, C3/C2 ratio, and total plasma HCY were statistically significant (*P* < 0.05). However, urinary methylmalonic acid values showed no statistical differences (*P* > 0.05). After treatment, these metabolic markers showed a remarkable tendency to decrease, with a significant statistical difference in both groups (*P* < 0.05). On comparing the degree of decline in all the above indicators after treatment, both a significant difference in plasma total HCY values and urinary methylmalonic acid levels could be identified (*P* < 0.05), while there was only a slight difference in the reduction in blood C3 values and C3/C2 ratios (*P* > 0.05). For patients carrying a c.482G > A mutation in a homozygous state or in compound heterozygosity, there was no statistical difference in the degree of decline in all the above biochemical indicators (*P* > 0.05).

**Table 3:** Biochemical results before and after treatment and the dosage of hydroxocobalamin of patients in different groups

Categories	Dosage of hydroxocobalamin (mg/kg/wk)	C3 (0.50–4.00 µmol/L)		C3/C2 (0.00–0.20)		Methylmalonic acid (0.2–3.6 µmol/L)		Total HCY (0.0–15.0 µmol/L)	
		Before treatment	After treatment	Before treatment	After treatment	Before treatment	After treatment	Before treatment	After treatment
c.482G > A group (*n* = 149)	0.30 (0.04–2.59)	5.64 (4.41–27.25)	2.21 (0.08–9.65)	0.49 (0.26–7.83)	0.09 (0.01–0.39)	86.15 (5.47–947.00)	2.36 (0.00–45.09)	68.20 (21.30–247.20)	13.36 (0.60–38.60)
Non-c.482G > A group (*n* = 150)	0.53 (0.06–4.31)	6.87 (4.69–28.43)	3.88 (0.06–10.45)	0.64 (0.27–9.40)	0.13 (0.01–0.41)	90.25 (10.60–1411.00)	10.22 (0.00–52.49)	96.30 (39.60–335.70)	30.40 (4.00–59.90)
*P*_1_	< 0.050	< 0.050	< 0.050	0.001	< 0.050	0.461	< 0.050	< 0.050	< 0.050
*P*_2_ (before–after)	/	< 0.050		< 0.050		< 0.050		< 0.050	
*P*_3_ (before–after)	/	< 0.050		< 0.050		< 0.050		< 0.050	
*P*_4_	/	0.530		0.410		< 0.050		< 0.050	

Data are presented as median (minimum–maximum). *P*_1_: the differences of the corresponding metabolites levels between c.482G > A group and non-c.482G > A group; *P*_2_: the differences of the corresponding metabolites levels between before treatment and after treatment in c.482G > A group; *P*_3_: the differences of the corresponding metabolites levels between before treatment and after treatment in non-c.482G > A group; *P*_4_: the differences of the degree of decline in the corresponding metabolites levels after treatment between c.482G > A group and non-c.482G > A group. *C2* acetylcarnitine, *C3* propionylcarnitine, *HCY* homocysteine. “/” no data

### Molecular genetic analysis

To better understand the genotype of the 195 patients carrying the c.482G > A variant in this study, we summarized their “another mutation” in another allele. Details are shown in Table 4. Biallelic mutations in the *MMACHC* gene were detected in 186/195 patients, while in the other nine cases, no mutations were detected in another allele except for the c.482G > A variant. Fifteen patients were homozygous for the c.482G > A variant and 171 were compound heterozygotes. As a result, 27 molecular variants in another allele of the *MMACHC* gene were identified, including nine frameshift mutations, eight nonsense mutations, five missense mutations, four deletion variations, and one splicing mutation. Among these variations, c.541G > T and c.435_436dup were novel. The five most prevalent variants were c.609G > A, c.658_660del, c.567dup, c.482G > A, and c.80A > G. The genotypes of all samples in the non-c.482G > A group are shown in Supplementary Table 1.

**Table 4:** Frequency of *MMACHC* variants in another allele in patients in c.482G > A group (*n* = 186)

Variation	Region	Nucleotide change	Amino acid change	Variation type	Variation frequency
1	E4	c.609G > A	p.W203*	Nonsense	62/186 (33.3)
2	E4	c.658_660del	P.K220del	Deletion	33/186 (17.7)
3	E4	c.567dup	p.I190Yfs*13	Frameshift	29/186 (15.6)
4	E4	c.482G > A	p.R161Q	Missense	15/186 (8.1)
5	E1	c.80A > G	p.Q27R	Missense	7/186 (3.8)
6	E2	c.217C > T	p.R73*	Nonsense	6/186 (3.2)
7	E4	c.445_446del	p.C149Hfs*32	Frameshift	4/186 (2.2)
8	E3	c.394C > T	p.R132*	Nonsense	3/186 (1.6)
9	E4	c.481C > T	p.R161*	Nonsense	3/186 (1.6)
10	E2	c.271dup	p.R91Kfs*14	Frameshift	2/186 (1.1)
11	E4	c.440G > A	p.G147D	Missense	2/186 (1.1)
12	E4	c.445_446insA	p.C149*	Nonsense	2/186 (1.1)
13	E4	c.448_449delATinsCC	p.I150P	Missense	2/186 (1.1)
14	E4	c.541G > T	p.D181Y	Missense	2/186 (1.1)
15	E4	c.615C > A	p.Y205*	Nonsense	2/186 (1.1)
16	E2	c.178dup	p.D60Gfs*18	Frameshift	1/186 (0.5)
17	E2	c.228_231del	p.D77Qfs*22	Frameshift	1/186 (0.5)
18	E3	c.315C > G	p.Y105X	Nonsense	1/186 (0.5)
19	E3	c.395_397del	p.R132del	Deletion	1/186 (0.5)
20	E3	c.398_399del	p.Q133Rfs*5	Frameshift	1/186 (0.5)
21	E4	c.435_436dup	p.S146Yfs*19	Frameshift	1/186 (0.5)
22	E4	c.561_572del	p.D188_A191del	Deletion	1/186 (0.5)
23	E4	c.626_627del	p.V209Dfs*35	Frameshift	1/186 (0.5)
24	E4	c.626dup	p.T210Dfs*35	Frameshift	1/186 (0.5)
25	E4	c.637G > T	p.E213*	Nonsense	1/186 (0.5)
26	IVS1	c.81 + 1G > A	/	Splicing	1/186 (0.5)
27	E1	Exon 1 del	/	Deletion	1/186 (0.5)

Data are presented as *n/n* (%). *MMACHC* methylmalonic aciduria and homocystinuria type C protein gene. “/” no data

### Follow-up and outcome evaluations

The clinical outcome evaluations of the biochemical results after treatment and the dosage of hydroxocobalamin at the last follow-up are shown in Table 3. The median dosage of hydroxocobalamin of patients in the c.482G > A group was significantly lower than that in non-c.482G > A group (*P* < 0.05). Furthermore, their metabolic conditions were better controlled after treatment, with much lower concentrations of blood C3, C3/C2 ratio, total plasma HCY, and urinary methylmalonic acid values. However, for patients carrying the c.482G > A mutation in a homozygous state or in compound heterozygosity, there was no statistical difference in the dosage of hydroxocobalamin (*P* > 0.05).

The clinical outcome evaluations of disease onset and basic motor function and language development are shown in Table 5. For individuals diagnosed through NBS or because of sibling MMA diagnosis, fewer cases developed clinical symptoms and more than 90% of cases remained with normal psychomotor and language development in the c.482G > A group. Compared with those in the non-c.482G > A group, the differences were statistically significant (*P* < 0.05). Similar results were found in patients diagnosed due to disease onset. In the c.482G > A group, only one neonatal-onset patient died at 4 months old because of refusal of treatment, 65.0% of patients had movement and/or speech impediments, while 33.3% of patients developed normally. In the non-c.482G > A group, four cases died of acute metabolic decompensation and/or encephalopathic crises induced by infection during follow-up, 85.5% patients had movement and/or speech development delay, while only 10.9% of cases developed normally. Compared with those in the non-c.482G > A group, individuals with a c.482G > A variation had significantly lower mortality and a higher proportion of patients with normal psychomotor and language development (*P* < 0.05). Compared with those diagnosed due to disease onset, individuals diagnosed with NBS in either group showed a better prognosis, with significantly lower mortality and a higher proportion of patients with normal psychomotor and language development (*P* < 0.05).

**Table 5:** Prognosis of psychomotor development of patients in different groups

Patients	Disease onset	Current psychomotor development status		Death
		Normal	Delayed	
c.482G > A group (*n* = 195)				
Diagnosed from newborn screening and sibling diagnosis (*n* = 125 + 10)	9 (6.7)	134 (99.3)	1 (0.7)	0 (0.0)
Diagnosed due to disease onset (*n* = 60)	60 (100.0)	20 (33.3)	39 (65.0)	1 (1.7)
All patients (*n* = 195)	69 (35.4)	154 (79.0)	40 (20.5)	1 (0.5)
Non-c.482G > A group (*n* = 200)				
Diagnosed from newborn screening (*n* = 90)	49 (54.4)	53 (58.9)	37 (41.1)	0 (0.0)
Diagnosed due to disease onset (*n* = 110)	110 (100.0)	12 (10.9)	94 (85.5)	4 (3.6)
All patients (*n* = 200)	159 (79.5)	65 (32.5)	131 (65.5)	4 (2.0)
Patients diagnosed from newborn screening and sibling diagnosis in different groups				
*χ*^2^	64.427	59.186	59.186	–
*P*	< 0.050	< 0.050	< 0.050	–
Patients diagnosed due to disease onset in different groups				
*χ*^2^	–	12.776	9.539	0.063
*P*	–	< 0.050	0.002	0.801
Whole patients in different groups				
*χ*^2^	78.739	84.338	79.442	0.760
*P*	< 0.050	< 0.050	< 0.050	0.383

Data are presented as *n* (%). “–” no data

At the final follow-up, within the c.482G > A group, none of the 69 symptomatic patients experienced vomiting, feeding difficulty, lethargy, coma, mental abnormalities, jaundice, or urinary incontinence. Out of the 35 patients who initially presented with lower limb weakness and poor exercise tolerance, 10 recovered, while 25 continued to exhibit limb weakness. Among the 26 patients with cognitive, learning, and memory decline at onset, eight returned to normal, and 18 experienced slow response and memory decline. Of the 25 patients with gait instability and abnormal posture at onset, only five recovered. Out of the 18 patients who had seizures at onset, 16 did not experience further seizures, while two patients, classified as having “secondary epilepsy,”, remained seizure-free after oral antiepileptic drug treatment. Among the three patients with hydrocephalus at onset, one underwent surgery and recovered well, while two patients continued to have mild hydrocephalus without surgery. Two patients with visual impairment maintained impaired vision but did not undergo regular fundus and visual acuity examinations. Of the three patients with abnormal renal function at onset, two recovered, and one still exhibited abnormal renal function with increased proteinuria and blood creatinine levels. One patient with pulmonary hypertension returned to normal.

Additionally, our cohort included 15 patients with homozygous c.482G > A mutations. Ten were diagnosed through NBS, two were identified following sibling onset confirmation and presented no onset or abnormal symptoms, and three were diagnosed after onset (with ages of onset at 3 months, 6 months, and 26 years, respectively). Among these, two patients were developmentally delayed, displaying abnormal walking posture, gait instability, and cognitive regression.

## Discussion

The CblC disease is more common in Chinese patients than in other populations. Affected individuals can present symptoms at any age ranging from prenatal to adulthood, and clinical manifestations vary from mild to life-threatening [14–16]. Patients with early-onset disease present symptoms in the first year of life, whereas patients with late-onset disease may have been previously asymptomatic and then suddenly develop variable symptoms under small stress events [3, 4]. Since the *MMACHC* gene was identified, numerous pathogenic variants have been reported. Several studies have suggested that there are some correlations between the phenotype and genotype [17, 18]. The c.482G > A (p.R161Q) variation seems to be related to late-onset disease [5–7]. This national multicenter retrospective study, therefore, aimed to elucidate the clinical features and outcome of 195 confirmed MMA patients with the *MMACHC* c.482G > A mutation, and to investigate the relationship between genotype and phenotype for the specific mutation c.482G > A.

In the present study, we investigated the number and onset age of symptomatic patients in the two groups. As expected, the majority of patients diagnosed through NBS or because of sibling MMA diagnosis in the c.482G > A group remained asymptomatic with pre-symptomatic treatment. For patients diagnosed by disease onset, the median onset age was 7 years and 6 months. In the non-c.482G > A group, most patients diagnosed either by NBS or disease onset developed clinical symptoms at a median age of before 1 month. This evidence further indicates that patients with the c.482G > A mutation show later onset. Nonetheless, the ages of onset for the three homozygous individuals were 3 months, 6 months, and 26 years, suggesting that the age of onset may not be associated with homozygous or heterozygous status.

In terms of the clinical symptoms, although the main clinical manifestations of patients in both groups were neurological impairment, acute metabolic decompensation, and acute encephalopathic crisis, presenting as lethargy, feeding difficulty, vomiting, and coma, these were more common in patients without the c.482G > A variant, while chronic neurological impairment, such as decline in cognitive, learning, and memory, gait instability and abnormal posture, psychiatric and behavioral disturbances, were more prevalent in patients with the c.482G > A variant (Table 2). These findings are consistent with previous reports [7, 19, 20]. Meanwhile, the initial clinical manifestations of all seven adult-onset patients in this study were psychiatric and behavioral disturbances. This suggests that although patients with the c.482G > A variant may have milder clinical symptoms and progress slowly, many individuals, especially with adult-onset cases, may present insidious and nonspecific symptoms and develop neurological disease in the absence of encephalopathic crises [21]. Interestingly, the incidence of ocular disease in this study is lower than that reported in other research [22]. As previously reported [22–24], ophthalmologic manifestations, including maculopathy, progressive retinal dysfunction, and, less frequently, optic atrophy could be seen in most patients with infantile CblC disease. In our cohort, only 11.9% of patients suffered from ocular disease in the non-c.482G > A group. For patients in the c.482G > A group, the incidence (2.9%) was much lower. However, it cannot be ruled out that some patients did not undergo a detailed ophthalmological examination. Therefore, more attention should be paid to the ophthalmological examination in future diagnosis and treatment. Moreover, there appears to be minimal variation in the spectrum of clinical symptoms at onset between patients identified through NBS and those diagnosed due to disease onset in both groups.

Regarding biochemical data in the c.482G > A group, the important diagnostic biomarkers, including blood C3, C3/C2 ratio, total plasma HCY, and urinary methylmalonic acid levels, were all lower than those in the non-c.482G > A group before treatment. Following treatment, even with a lower dosage of hydroxocobalamin, these diagnostic biomarker concentrations in individuals with the c.482G > A mutation decreased to a level closer to normal, and more than half the patients even got normal total plasma HCY and urinary methylmalonic acid levels. These results serve to illustrate that patients carrying a c.482G > A variation had a milder biochemical phenotype and better response to hydroxocobalamin than those without. It has been proposed that the milder biochemical phenotype could be explained by findings about the specific c.482G > A mutant protein, including the higher expression, the intermediate stability, and being easier to become stable by an increased cobalamin concentration [17, 25, 26].

It is noteworthy that different genotypes seem to have different impacts on the onset of this disorder as well as its prognosis [17]. Compared to previous studies [1, 7, 20], the proportion of mortality in our study is obviously lower. In comparison with individuals in the non-c.482G > A group, those who carried the c.482G > A variation were more likely to remain asymptomatic and have better clinical outcomes in both biochemical metabolites control and psychomotor development. Especially for individuals diagnosed through NBS and because of sibling CblC disease diagnosis, with pre-symptomatic treatment, more than 90% of patients carrying the c.482G > A mutation remained asymptomatic, and almost all had normal psychomotor development as of this writing. For patients in the non-c.482G > A group diagnosed through NBS, 41% still experienced psychomotor delay despite prompt treatment, likely due to early damage sustained during the fetal period.

Furthermore, we found that in both the c.482G > A and non-c.482G > A groups, patients identified via NBS exhibited fewer symptoms and a lower incidence of psychomotor delay compared with those diagnosed after disease onset, potentially attributable to timely diagnosis and treatment. Conversely, among patients diagnosed due to disease onset, 65% and 85.5% of cases in the two groups, respectively, ultimately experienced delayed motor function and language development, even with proper treatment. This suggests that NBS, which enables pre-symptomatic treatment, may offer protective effects on the prognosis of children with CblC defects, particularly for patients carrying the c.482G > A mutation, as indicated in our previous study [27]. However, the benefits of NBS are challenging to assess accurately due to the limited follow-up period. On the other hand, smaller sample sizes may imply more severe sampling bias. To elucidate the true outcomes, it is necessary to collect additional samples and conduct long-term follow-up studies.

Furthermore, even though the c.482G > A variation was associated with late-onset and milder phenotypes of CblC disease, the incidence of neurological complications was still high without pre-asymptomatic treatment and once the disease occurred. Consistent with previous reports [1], a delay in treatment may lead to irreversible damage. It cannot be overlooked that one of our patients was found to have developmental delay at the age of 17 months, due to the refusal of confirmative testing and pre-symptomatic treatment. Therefore, if affected patients can be diagnosed before symptomatic onset, accept the timely therapy, and then pay more attention to avoid trigger factors such as respiratory or digestive tract infections, vaccination, trauma, and surgery, it may be possible to prevent the disease onset and metabolic crises. NBS is currently the main method of pre-symptomatic diagnosis for CblC disease [28, 29]. However, it was reported that some patients with the c.482G > A mutation and other late-onset CblC disease may be missed through the current NBS program [5, 30]. We believe that the combination of rapid and accurate next-generation sequencing technology and biochemical screening may improve NBS efficiency of late-onset CblC disease in the future [31–33].

This study has several limitations. CblC disease is a multi-organ and multi-system disorder. It is essential to conduct ophthalmological, renal, neurological, hematological, and, in some cases, cardiopulmonary assessments during follow-up. While we performed a systematic evaluation of these aspects at the patients’ onset, we were unable to obtain comprehensive clinical information during follow-up. This is because our study involved numerous hospitals with varying levels of follow-up. Furthermore, some patients were followed up through telephone surveys, preventing a complete assessment at their last follow-up. All subjects will continue to be monitored to gather more information about the disease. In future follow-up sessions, we will employ patient recall to conduct a more comprehensive evaluation and study.

In conclusion, this study represented the largest Chinese patient cohort of CblC disease with the *MMACHC* c.482G > A mutation. We further proved that the c.482G > A pathogenic variant is associated with late-onset and milder phenotypes of CblC disease. Patients carrying this mutation tend to have a relatively better response to hydroxocobalamin, better metabolic control, and more favorable neurological outcomes. NBS and appropriate pre-symptomatic treatment seem to be helpful in early diagnosis, resulting in favorable clinical outcomes. This study explored the variable clinical manifestation spectrum and outcome of patients with the *MMACHC* c.482G > A variant to some extent, which may be helpful for a more comprehensive and systematic understanding of this patient population.

## Supplementary Information

Below is the link to the electronic supplementary material.

Supplementary file 1 (PDF 77 KB)

## Data Availability

The data sets generated during and/or analyzed during the current study are available from the corresponding author on reasonable request.
