# Supplementary material for: Variable phenotypes and outcomes associated with the MMACHC c.482G > A mutation: follow-up in a large CblC disease cohort
Source: World J Pediatr. 2023 Dec 9;20(8):848–58. doi: 10.1007/s12519-023-00770-2 (PMC11402842; doi:10.1007/s12519-023-00770-2)
Supplement: Supplementary file 2 — Supplementary file 1 (PDF 77 KB) [file 12519_2023_770_MOESM2_ESM.pdf]

Supplementary Table 1. Genotype of all samples in the non-c.482G>A group

Variation	Region	Nucleotide change	Amino-acid change	Variation type	Mutation alleles	Variation frequency
1	E4	c.609G>A	p.W203*	Nonsense	135	135/395 (34.2)
2	E4	c.658_660del	P.K220del	Deletion	62	62/395 (15.7)
3	E4	c.567dup	p.I190Yfs*13	Frame-shift	36	36/395 (9.1)
4	E1	c.80A>G	p.Q27R	Missense	32	32/395 (8.1)
5	E3	c.394C>T	p.R132*	Nonsense	15	15/395 (3.8)
6	E3	c.315C>G	p.Y105X	Nonsense	13	13/395 (3.3)
7	E2	c.217C>T	p.R73*	Nonsense	11	11/395 (2.8)
8	E1	c.1A>G	p.M1V	Missense	10	10/395 (2.5)
9	E4	c.445_446del	p.C149Hfs*32	Frame-shift	8	8/395 (2.0)
10	E3	c.365A>T	p.H122L	Missense	7	7/395 (1.8)
11	E3	c.331C>T	p.R111*	Nonsense	6	6/395 (1.5)
12	E4	c.626_627del	p.V209Dfs*35	Frame-shift	6	6/395 (1.5)
13	E4	c.615C>A	p.Y205*	Nonsense	5	5/395 (1.3)
14	E4	c.626dup	p.T210Dfs*35	Frame-shift	5	5/395 (1.3)
15	E1	Exon 1 del	/	Deletion	4	4/395 (1.0)
16	IVS 2	c.276+2T>C	/	Splicing	3	3/395 (0.8)
17	E4	c.481C>T	p.R161*	Nonsense	3	3/395 (0.8)
18	E4	c.666C>A	p.Y222*	Nonsense	3	3/395 (0.8)
19	E4	c.541G>T	p.D181Y	Missense	2	2/395 (0.5)
20	E1	c.104T>G	p.L35W	Missense	1	1/395 (0.3)
21	E1	c.125C>T	p.P42L	Missense	1	1/395 (0.3)
22	E1	c.146_154del	p.49_51del	Deletion	1	1/395 (0.3)
23	E2	c.271A>T	p.R91*	Nonsense	1	1/395 (0.3)
24	E2	c.275_276delAG	p.S93Pfs*11	Frame-shift	1	1/395 (0.3)
25	E3	c.311T	p.R111*	Nonsense	1	1/395 (0.3)
26	E3	c.321G>A	p.V107V	Missense	1	1/395 (0.3)
27	E3	c.349C>T	p.R132*	Nonsense	1	1/395 (0.3)
28	E3	c.374G>A	p.G218N	Missense	1	1/395 (0.3)
29	E3	c.388T>C	p.Y130H	Missense	1	1/395 (0.3)
30	E3	c.393_395delACG	p.132_132del	Deletion	1	1/395 (0.3)
31	E3	c.398_399delAA	p.Q133Rfs*5	Frame-shift	1	1/395 (0.3)
32	E3	c.427C>T	p.Q143*	Nonsense	1	1/395 (0.3)
33	E4	c.445T>C	c.C149R	Missense	1	1/395 (0.3)
34	E4	c.448_449AT>CC	p.I150P	Missense	1	1/395 (0.3)
35	E4	c.449T>G	p.I150R	Missense	1	1/395 (0.3)
36	E4	c.452A>G	p.H151R	Missense	1	1/395 (0.3)
37	E4	c.455_457del	p.P152del	Deletion	1	1/395 (0.3)
38	E4	c.457C>T	p.R153*	Nonsense	1	1/395 (0.3)
39	E4	c.467G>A	p.G156D	Missense	1	1/395 (0.3)
40	E4	c.599G>A	p.W200*	Nonsense	1	1/395 (0.3)
41	E4	c.616C>T	p.R206W	Missense	1	1/395 (0.3)

42	E4	c.616delC	p.R206Gfs*4	Frame-shift	1	1/395 (0.3)
43	E4	c.617G>A	R206Q	Missense	1	1/395 (0.3)
44	E4	c.651_653del	p.E218del	Deletion	1	1/395 (0.3)
45	E4	c.689G>A	p.R230Q	Missense	1	1/395 (0.3)
46	IVS 1	c.81+1G>A	/	Splicing	1	1/395 (0.3)
47	E1	c.89G>A	p.W30*	Nonsense	1	1/395 (0.3)
48	E1	c.90G>A	p.W30*	Nonsense	1	1/395 (0.3)
